# Effect of NaOCl on cyclic fatigue resistance

**DOI:** 10.6026/973206300191348

**Published:** 2023-12-31

**Authors:** Akshayraj Langaliya, Kanika Singh Dhull, Bharti Gupta, Muhammad Atif Saleem Agwan, Shekhar Gupta, Yamuna Macha, Ramanpal Singh Makkad

**Affiliations:** 1 Department of Conservative Dentistry and Endodontics, AMC Dental College and Hospital, Gujarat, India; 2 Department of Pedodontics and Preventive Dentistry, Kalinga Institute of Dental Sciences, KIIT Deemed to be University, Bhubaneswar, Odisha, India; 3 Department of Maxillofacial Surgery and Diagnostic Sciences, College of Dentistry, Jazan University, Jazan 45142, Saudi Arabia; 4 Department of Restorative Dentistry in Ar-Rass, Qassim University, Kingdom of Saudi Arabia; 5 Department of Prosthodontic Dental Sciences, College of Dentistry, Jazan University, Jazan 45142, Saudi Arabia; 6 Department of Oral Pathology and Microbilogy, H.K.E.S' S N Dental College and Hospital, Kalburgi, India; 7 Department of Oral medicine and Radiology, New Horizon Dental College and Research, Institute, Sakri, Bilaspur, Chhattisgarh, India

**Keywords:** Cyclic fatigue, TruNatomy, HeroGold, Protaper Gold, resistance to cyclic fracture

## Abstract

The impact of 5.25 percent NaOCL on the resistance offered by TruNatomy, Hero Gold and ProtaperGold against cyclic fracture as against the treatment of NiTi rotary files with distilled water is of interest to dentists. Inside the stainless steel blocks, man-made canals were created for the purpose of testing the cyclic fatigue. 60 endodontic files were taken as study specimens. It was observed that values of number of cycles to fracture in NaOCl among three file systems were in the order of TruNatomy (1053.50 ± 134.81)>Hero Gold (652.66 ± 58.66) > ProtaperGold (494.50 ± 47.69). The TruNatomy file system reflected greatest cyclic fatigue resistance. It was also found that cyclic fatigue resistance in NiTi rotary files studied here is not hampered by 5.25% NaOCl.

## Background:

The use of nickel-titanium (NiTi) alloys to be the ideal material for being utilised in the production of rotary instruments in endodontics has split the evolution of endodontics into two distinct periods. Since its inception, processes of instrumentation of root canals in endodontics have undergone such significant changes that it is now regarded as an advancement in technology that marked the starting point of modern endodontics [1-2]. The instruments employed during endodontic treatments are mostly made using these NiTi alloys [3-4]. Nevertheless, because it compromises the effectiveness of endodontic treatment, fracture of NiTi alloy-based endodontic instruments is a prevalent issue these days [5-6]. One of the main causes of endodontic instrument breaking and after that instrument failure is thought to be cyclic fatigue. Whenever endodontic rotary instruments are turned in a curved root canal, cyclic fatigue typically occurs [7-8]. It causes cycles of the compression or tension to develop in a recurring pattern. A drop in temperature and the imposition of stress cause phase conversion to occur in these NiTi instruments [9-10, 11,12]. Several manufacturing process improvements have been performed to increase the resistance to breakage of rotary instruments based on NiTi alloys [13- 14].

Several heat treatments and adjustments to the internal instrument geometry were among these enhancements. Numerous rotary endodontic instruments made up of NiTi alloys have been released [15-16]. ProTaper Gold NiTi rotary files are made using the geometrical characteristics of ProTaper Universal endodontic instruments. However, because of the sophisticated heat treatment-based process of manufacturing, ProTaper Gold instruments have better metallurgical properties [17-18]. Consequently, ProTaper Gold instruments exhibit higher resistance towards cyclic fatigue and increased flexibility when compared against Protaper Universal instruments [19-20,21, 22]. In order to increase the resistance to cyclic fatigue, TruNatomy files were recently introduced. There are three different sizes accessible: small size, medium size, and prime size [23, 24]. It comes in an off-centered parallelogram cross-section layout with an adaptable taper geometry. In light of the metallurgical phase transition from austenite to martensite, the heat treatment increases the elasticity as well as flexibility [25-26].

It has been highly advised that the endodontic root canal during biomechanical preparation should be actively rinsed with some endodontic irrigants. Sodium hypochlorite (NaOCl) is widely utilized for this purpose [8]. It is believed to possess vital abilities to dissolve organic tissue as well as antibacterial properties. NaOCl is used in a variety of concentrations varying from 0.5 percent and extending up to 5.25%. It has been suggested that the concentration of the adjacent medium may affect how tired rotary endodontic instruments become [9]. Until now, no research has been done on how 5.25% sodium hypochlorite affects the cyclic fatigue of above discussed nickel-titanium alloys [10]. Therefore, it is of interest to evaluate and compare the impact of 5.25 percent NaOCL on the resistance offered by TruNatomy, Hero Gold and ProtaperGold against cyclic fracture and comparing these results with that of results obtained on the treatment of NiTi rotary files with distilled water.

## Methods and Materials:

## Sample size calculation:

Information from the earlier study [Alfawaz H et al. Journal of Endodontics. 2018; 44(10): 1563-156 shows the expected pooled standard deviation of push-out bond strength as 253.10 and a mean difference of 300.7. Utilising the formula, there was calculation a total sample size.. The sample size was calculated for an alpha error of 5% and statistical power at 95%. The sample size determined was n =20. Hence, there will be n/2 = 10 samples in each group. Total sample size was 60.

## Methodology:

It was an in vitro observational study. 60 endodontic files were taken as study specimens for this study. These instruments were randomly distributed in three main categories and 6 subgroups (Table 1).

Every instrument's length was maintained at 25 mm. Prior to the study, the instruments were examined under high magnification for any defects and deformities. Nevertheless, following the assessment, no instruments were eliminated. Inside the stainless steel blocks, man-made canals were created for the purpose of testing the cyclic fatigue. A laser-based micro-machine was used to prepare these artificial root canals. The artificial canals' dimensions were maintained in accordance with the file's width. It measured 0.1 mm more than the instrument's width. The canals' curvature angle was maintained at 60 degrees, its radius of curvature was maintained at 5 millimeters, and its centre of curvature was located 5 millimeters from the instrument's tip. To prevent the instrument from slipping and to confirm the exact moment of the instrument's fracture, glass was put in place to safeguard the artificial canal.

The apparatus containing the artificially canalized stainless steel blocks was filled with a mixture of 5.25% NaOCl and distilled water for experimentation. A temperature adjustment of 25 degrees Celsius was made. Following the manufacturer's instructions, the instrument was rotated using rotary handpiece inside the man-made canal. (300rpm speed/ 3 Nmm torque for Protaper Gold, 350rpm speed/3 Nmm torque for TruNatomy files, and 400rpm speed/1.2Nmm torque for Hero Gold).The instrument was turned until a fracture occurred. Seconds were recorded as the duration of fracture of instruments. Using the following formula, the number of cycles to fracture (NCF) was examined. In order to reduce friction, special high-flowing synthetic oil was utilized.

NCF = time to fracture x rpm/60.

The same clinician performed every experimental procedure. As soon as any signs of corrosion that could result from NaOCl's corrosive activity appeared, the artificial canal was replaced. An electronic digital caliper was used to measure the size of the fractured fragment. From each group, two instruments with fractures were chosen. Following a thorough cleaning in an ultrasonic bath containing pure alcohol, the surfaces near the fracture were inspected under a scanning electron microscope (EVOLS10, ZEISS) at magnifications of 300x and 2000x.

## Statistical analysis:

Excel (Microsoft, Seattle, WA, USA) and SPSS (Version 17.0.1, Armonk, NY, USA) were the statistical software packages used for the analyses. The means and standard deviations (SD) of the no cycles for fracture in each of the categories were computed as descriptive statistics. The Chi-Squared test was used to examine resistance offered against cyclic fatigue of each category. The variations among categories were calculated using the Mann-Whitney U test. The differences in number of cycles for fracture between the three categories were assessed using the Kruskal-Wallis test. P<.05 was chosen as the significance threshold for all statistical tests.

## Results:

Cyclic fatigue decreased as the NCF increased and vice versa. When there was an analysis of NCF in the TruNatomy group then it was observed that the values of NCF were 1153.83 ± 122.39 when distilled water was used as an endodontic irrigant. It was decreased to 1053.50 ± 134.81 when sodium hypochlorite solution was used as an endodontic irrigant. However, this difference was not significant statistically (p>0.05).

On carrying out such analysis in Protaper Gold, it was noticed that values of NCF were 505.00 ± 48.13 when distilled water was used as an endodontic irrigant. It decreased to 494.50 ± 47.69 when sodium hypochlorite solution was used as an endodontic irrigant. However, this difference was not significant statistically (p>0.05). When such analysis was carried out in HeroGold, it was noticed that the values of NCF were 657.99 ± 52.40 when distilled water was used as an endodontic irrigant. It decreased to 652.66 ± 58.66 when sodium hypochlorite solution was used as an endodontic irrigant. However, this difference was not significant statistically (p>0.05). It was observed that there was rise in cyclic fatigue when NaOCl was incorporated as an endodontic root canal irrigant in place of incorporating distilled water. The change was not significant statistically (p>0.05) as shown in Table 2.

On carrying out a comparison among the three groups of instruments, it was observed that values of NCF in distilled water among three file systems were in the order of TruNatomy (1153.83 ± 122.39)>Hero Gold (657.99 ± 52.40)> ProtaperGold (505.00 ± 48.13). The difference was significant statistically (p=0.001). It was observed that values of NCF in NaOCl among three file systems were in the order of TruNatomy (1053.50 ± 134.81)>Hero Gold (652.66 ± 58.66) > ProtaperGold (494.50 ± 47.69). The difference was significant statistically (p=0.001) (Table 2).

SEM cross-section pictures in 300X magnification, 1000X magnification, and 2000X magnification, as well as a surface view in 300X magnification, indicate crack initiation, concentric abrasive imprints, indentation points, fatigue stippled zone, and overload rapid fracture area at the center of rotation.. The instruments' shattered cross-sections revealed that cracks began at the cutting edge of the tool. On the fracture surfaces, there were minute depressions. In distilled water or 5.25 per cent sodium hypo-chloride, the files revealed no pitting or fissure corrosion.

## Discussion:

Cyclic fatigue is one of the most frequent causes of NiTi instrument fractures [13- 14]. Consequently, it is critical from a clinical standpoint to examine the effects of various irrigants, such as distilled water and sodium hypochlorite solution at 5.25%, on the ability of rotating nickel-titanium alloys to withstand cyclic fatigue [16-17]. This study was conducted to evaluate and compare the impact of 5.25 percent NaOCL on the resistance offered by TruNatomy, Hero Gold and ProtaperGold and comparing these results with that of results obtained on the treatment of NiTi rotary files with distilled water.

Data are similar to study conducted earlier where TruNatomy files showed highest resistance against cyclic fracture. In other study; it was observed that NiTi files cyclic resistance is not significantly reduced with exposure to NaOCl [12-15]. Compared to traditional NiTi alloys, the controlled memory heat treatment of TruNatomy files aided in the preservation of canal curvatures more effectively [16- 18]. By altering the transition temperature and microstructure of the alloys as well as the crystal lattice arrangement, thermal heat treatment of the NiTi alloys increased their flexibility and fatigue resistance. The file usually exhibits softness and ductility during the martensitic phase. It makes the resistance to fracture higher [19- 21].

In a related study, authors assessed the impact of varying sodium hypochlorite concentrations at varying temperatures in nickel-titanium alloy-based rotary instruments. Their data were in contrast to those of our current investigation [13- 15]. They came to the conclusion that rotary instruments based on nickel-titanium alloys are prone to fracture due to cyclic fatigue when exposed to NaOCl (5.25%) [16,18, 21,23]. That study's file systems, however, were not the same as ours. In a study to assess sodium hypochlorite's impact on nickel-titanium alloys' cyclic fatigue authors discovered that sodium hypochlorite impairs the alloys' ability to withstand cyclic fatigue [11,14]. These outcomes run counter to what the current study found.

The cyclic fracture resistance in NiTi alloy based rotary instruments is decreased by NaOCl incorporated in different concentrations, according to studies elsewhere [16,18,23- 25]. NaOCl did not, however, have any such effect on the cyclic fatigue resistance in our study. The differences in methodology may account for some of the variations in the study results. In these investigations, the instruments under investigation were submerged in solutions containing sodium hypochlorite for an extended period of time, up to two hours, and at temperatures as high as 60 degrees Celsius. These circumstances weren't representative of the real clinical circumstances. Conversely, in order to approximate clinical settings, this investigation entailed dynamically immersing only the 16 mm working portion of endodontic NiTi rotary instruments of three distinct varieties, based on torque and speed, in 5.25% NaOCl solution for five minutes at room temperature, as directed by the manufacturer. In order to maintain an achievable time frame for clinical practice, the duration of contact of the instrument with the solution was determined to be 5 minutes [17-20]. Therefore, in contrast to these earlier investigations, Data regarding the influence of NaOCl on cyclic fracture resistance in NiTi alloy based rotary endodontic instruments were not statistically significant, despite the fact that surface alterations were observed in the instruments under investigation.

Sodium hypochlorite has been shown to be able to remove nickel from an instrument's surface, resulting in micro-pitting that may be detrimental to the nickel-titanium rotary file's properties [12,14]. The development of corrosion zones and a decrease in the resistance of nickel-titanium alloy instruments against cyclic fatigue occur on treatment of NiTi files with NaOCl [17-20]. The cross-section and design offer superior resistance against cyclic fatigue in TruNatomy files. HeroGold instruments had more resistance gainst cyclic fatigue than ProtaperGold instruments, but not as much as TruNatomy instruments. The manufacturers claim that these files have adjustable pitch and helical angulation that advance in tandem with the endodontic instrument's taper. The endodontic instrument cannot twist thanks to its redesigned construction [23,24, 27,28,29]. One of the study's strengths was that the artificial canals' dimensions were maintained in accordance with the measurements of the instruments in the group under analysis. To stay within the realistic time frame for clinical practice, other strengths encompassed the application of the scientifically accepted state of immersion of only the component that works of endodontic NiTi rotary file instruments in 5.25% NaOCl solution for 5 minutes at room temperature. This study has limitations. Instead of using a real root canal, the study was conducted in artificial canals with mechanical designs. Debris generated throughout biomechanical preparation in the natural root canal can have an impact on cyclic fatigue. This study did not address this aspect of debris.

## Conclusion:

Data shows that the TruNatomy file system reflected greatest cyclic fatigue resistance. It was also found that cyclic fatigue resistance in NiTi rotary files studied here is not hampered by 5.25% NaOCl statistically. Some alterations were observed at the surface of NiTi rotary files after being treated with 5.25% sodium hypochlorite irrigant solution. Hence, sodium hypochlorite (5.25%) is useful for irrigation of root canals.

## Figures and Tables

**Table 1:** Distribution of study specimens in different groups

**Main groups**	**File system**	**Subgroups**	**Irrigant solution**	**Number (n)**
Category A	TruNatomy	A1	5.25% NaOCl	10
		A2	Distilled water	10
Category B	Protaper Gold	B1	5.25% NaOCl	10
		B2	Distilled water	10
Category C	Hero Gold	C1	5.25% NaOCl	10
		C2	Distilled water	10

**Table 2:** Comparison of cyclic fatigue

**Groups**	**Protaper Gold (PTG)**	**Hero Gold (HG)**	**TruNatomy (TN)**	**p-value**	**PTG vs HG**	**PTG vs TN**	**HG vs TN**
DW	505.00 ± 48.13	657.99 ± 52.40	1153.83 ± 122.39	0.001*	0.001*	0.001*	0.001*
NaOCl	494.50 ± 47.69	652.66 ± 58.66	1053.50 ± 134.81	0.001*	0.001*	0.001*	0.001*
Difference	10.5	5.33	100.33	--	--	--	--
p-value	0.791 (NS)	0.820 (NS)	0.095 (NS)	--	--	--	--
* indicates significant difference at p ≥ 0.05; NS: Non-significant difference; PTG: Protaper Gold, HG: Hero Gold, TN: TruNatomy
